# Co-infection of mice with SARS-CoV-2 and *Mycobacterium tuberculosis* limits early viral replication but does not affect mycobacterial loads

**DOI:** 10.3389/fimmu.2023.1240419

**Published:** 2023-09-01

**Authors:** Paul J. Baker, Eduardo P. Amaral, Ehydel Castro, Andrea C. Bohrer, Flor Torres-Juárez, Cassandra M. Jordan, Christine E. Nelson, Daniel L. Barber, Reed F. Johnson, Kerry L. Hilligan, Katrin D. Mayer-Barber

**Affiliations:** ^1^ Inflammation and Innate Immunity Unit, Laboratory of Clinical Immunology and Microbiology, National Institute of Allergy and Infectious Diseases (NIAID), National Institutes of Health (NIH), Bethesda, MD, United States; ^2^ T Lymphocyte Biology Section, Laboratory of Parasitic Diseases, NIAID, NIH, Bethesda, MD, United States; ^3^ SARS-CoV-2 Virology Core, Laboratory of Viral Diseases, NIAID, NIH, Bethesda, MD, United States; ^4^ Immunobiology Section, Laboratory of Parasitic Diseases, NIAID, NIH, Bethesda, MD, United States

**Keywords:** lung, mycobacterium tuberculosis, SARS-CoV-2, tuberculosis, COVID-19, Type-I interferon, co-infection

## Abstract

Viral co-infections have been implicated in worsening tuberculosis (TB) and during the COVID-19 pandemic, the global rate of TB-related deaths has increased for the first time in over a decade. We and others have previously shown that a resolved prior or concurrent influenza A virus infection in *Mycobacterium tuberculosis* (*Mtb*)-infected mice resulted in increased pulmonary bacterial burden, partly through type I interferon (IFN-I)-dependent mechanisms. Here we investigated whether SARS-CoV-2 (SCV2) co-infection could also negatively affect bacterial control of *Mtb*. Importantly, we found that K18-hACE2 transgenic mice infected with SCV2 one month before, or months after aerosol *Mtb* exposure did not display exacerbated *Mtb* infection-associated pathology, weight loss, nor did they have increased pulmonary bacterial loads. However, pre-existing *Mtb* infection at the time of exposure to the ancestral SCV2 strain in infected K18-hACE2 transgenic mice or the beta variant (B.1.351) in WT C57Bl/6 mice significantly limited early SCV2 replication in the lung. *Mtb*-driven protection against SCV2 increased with higher bacterial doses and did not require IFN-I, TLR2 or TLR9 signaling. These data suggest that SCV2 co-infection does not exacerbate *Mtb* infection in mice, but rather the inflammatory response generated by *Mtb* infection in the lungs at the time of SCV2 exposure restricts viral replication.

## Introduction

Pulmonary viral infections have been shown to both increase the likelihood and exacerbate the severity of secondary bacterial infections in the lung (1–7). The underlying immunological mechanisms are diverse and range from lung epithelial barrier breakdown and augmented adhesion of pathogens to the subversion of both adaptive and innate immunity from protective anti-bacterial pathways towards detrimental anti-viral inflammatory pathways like type-I interferon (IFN-I) (5, 8). Viral co-infections also play a role in the exacerbation of *Mycobacterium tuberculosis* (*Mtb*) infection (9), one of the leading causes of infectious disease-related mortality worldwide (10). For example, co-infection with cytomegalovirus (CMV) has been associated with an enhanced risk of tuberculosis (TB) disease (11–13). Furthermore, there are marked associations between influenza A virus (IAV) co-infection at the time of TB diagnosis and elevated *Mtb* burden (14), as well as increased risk of mortality in TB patients co-infected with both *Mtb* and IAV (15). *Mtb*-infected mice that were either simultaneously or subsequently infected with murine pneumonia virus (PVM) or IAV have been shown to have exacerbated lung tissue pathology (16). Our previous work demonstrated that simultaneous or prior IAV co-infection elevates pulmonary *Mtb* bacterial burden and reduces host survival after *Mtb* infection (17, 18). When IAV infection coincided with initial priming of *Mtb*-specific T cell responses, loss of bacterial control was dependent on elevated IFN-I and interleukin-10 (IL-10) signaling ultimately resulting in a reduced *Mtb*-specific CD4^+^ T cell response (17, 18).

Since the beginning of the COVID-19 pandemic, caused by SARS-CoV-2 (SCV2), TB diagnosis and case reporting reduced globally by 18% despite no change in the actual incidence of TB infection (10, 19). Importantly, a 7.5% increase in global TB deaths was observed, marking the first year-on-year increase in the global TB death toll since 2005 (10). A clear understanding of whether co-infection with SCV2 and *Mtb* has immunological consequences on the outcome of TB or COVID-19 is confounded by non-biological factors of the COVID-19 pandemic, including reduced BCG vaccination rates, disrupted TB outreach services and amplified global poverty (20). In addition, there have been reduced rates of early TB diagnosis during the COVID-19 pandemic attributed to reduced availability of staff and equipment for clinics and diagnostic labs (20–23) and reduced patient presentation due to fear of COVID-19 infection or increased social stigma around respiratory symptoms (24). TB treatment regimens, which already faced significant challenges before the pandemic because of the intensive and prolonged course of antibiotics required, have also been negatively impacted in TB-endemic countries during the pandemic (23, 25–27). Alongside negative TB outcomes, clinical reports have shown that *Mtb* and SCV2 co-infection results in a greater likelihood of severe COVID-19 disease (by an odds ratio of 2.21), COVID-19-related death (by an odds ratio of 2.77) (28) and overall elevated risk of negative clinical outcomes in co-infected individuals (29). Mechanistic studies into the possibility of immunological interactions critically influencing the outcome of *Mtb* and SCV2 co-infections are needed to develop effective strategies to reduce the mortality rate of both diseases (30, 31).

To directly ask whether co-infection with *Mtb* and SCV2 has a biological impact on the outcome of either TB or COVID-19, we sequentially infected mice with SCV2 followed by *Mtb* or co-infected *Mtb*-infected mice with SCV2. We show here that regardless of the order of infection co-infection with SCV2, unlike co-infection with IAV, does not alter the outcome of *Mtb* infection in mice. Moreover, we show that early pulmonary SCV2 replication is suppressed in chronically *Mtb*-infected mice through a mechanism that is dependent on mycobacterial dose but does not require signaling through type-I interferon (IFN-I) or toll-like receptor 2 (TLR2) or TLR9.

## Results

### Infection of mice with SCV2 one month before *Mtb* exposure does not alter pulmonary *Mtb* burden or pathology

**Figure 1:**
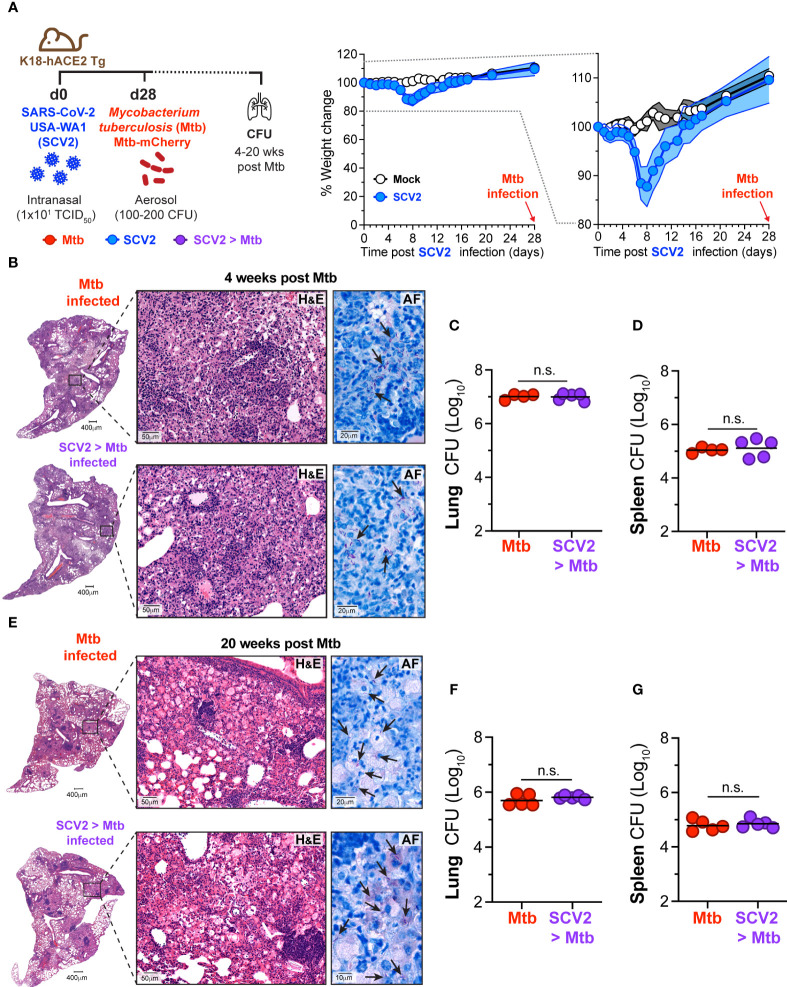
SCV2 infection one month before Mtb infection does not exacerbate Mtb disease. **(A)** Left: Schematic of experimental set-up where K18-hACE2 Tg mice were infected intranasally with 10 TCID_50_ SCV2 (USA-WA1/2020) or mock supernatant 28 days before aerosol infection with 100 – 200 CFU *Mtb* and mice were euthanized either 4 or 20 weeks after *Mtb* infection. Middle: Weight loss of K18-hACE2 Tg mice after SCV2 infection and before *Mtb* infection Right: Selected range of weight change curve to highlight differences in weight loss between SCV2 and Mock infected groups (n= 4-5 per group from one experiment representative of two independent experiments, mean ± SD as traveling error bars). **(B)** Representative hematoxylin and eosin (H&E) and acid-fast AF staining of lung tissue from mice at 4 weeks post *Mtb* infection, with or without prior SCV2 infection (arrows indicate examples of *Mtb* bacteria, scale bars indicate magnification). **(C, D)** *Mtb* CFU in **(C)** lungs and **(D)** spleens of mice at 4 weeks post *Mtb* infection. **(E)** Representative H&E and AF staining of lung tissue 20 weeks post *Mtb* infection with and without prior SCV2 infection (arrows indicate examples of *Mtb* bacteria, scale bars indicate magnification). **(F, G)** *Mtb* CFU in **(F)** lungs and **(G)** spleens of mice at 20 weeks post *Mtb* infection (n= 4-5 per group from one independent experiment per timepoint, geometric mean, two-tailed Mann Whitney test). n.s. = not significant.

We have previously shown that in mice, prior IAV infection leads to elevated pulmonary bacterial burden 16 weeks following subsequent *Mtb* infection (17). To determine whether prior infection with SCV2 similarly impacts the outcome of *Mtb* infection we used human Angiotensin Converting Enzyme 2 transgenic (K18-hACE2 Tg) mice, which are susceptible to infection with the ancestral strain of SCV2. K18-hACE2 Tg mice were infected with a sub-lethal dose of the hCoV-19/USA-WA1/2020 (USA-WA1/2020) isolate of SCV2 and 28 days later infected with *Mtb* (**Figure 1A**). *Mtb* disease was allowed to develop, and lungs and spleens were collected at 4 weeks (**Figures 1B–D**) or 20 weeks (**Figures 1E–G**) post *Mtb* infection. SCV2 infection resulted in transient weight loss 5 – 8 days post infection (**Figure 1A**). Lung pathology or bacterial distribution was determined by hematoxylin and eosin (H&E), and acid fast (AF) staining of lung sections 4 weeks post-*Mtb* infection, however no difference was detected when comparing mice previously infected with SCV2 to those animals that received *Mtb* alone (**Figure 1B**). Importantly, pulmonary (**Figure 1C**) or splenic (**Figure 1D**) bacterial loads were unchanged in mice previously infected with SCV2 compared to *Mtb*-only mice. To test whether prior SCV2 infection may affect the control of *Mtb* at a later timepoint, we assessed lung pathology and bacterial burden at 20 weeks post-*Mtb* infection. Again, H&E and AF staining did not reveal changes in lung pathology or bacterial localization between mice with prior SCV2 infection compared to mice that were only *Mtb* infected (**Figure 1E**). Similarly, previous SCV2 infection did not alter pulmonary (**Figure 1F**) or splenic (**Figure 1G**) bacterial loads at this later 20-week timepoint. Taken together, and in contrast to findings with prior IAV infection (17), our data here suggest that prior infection with SCV2 does not lead to increased *Mtb*-driven disease or impairment of *Mtb* bacterial replication in mice.

### Co-infection with SCV2 does not affect *Mtb* burden or lung pathology

**Figure 2:**
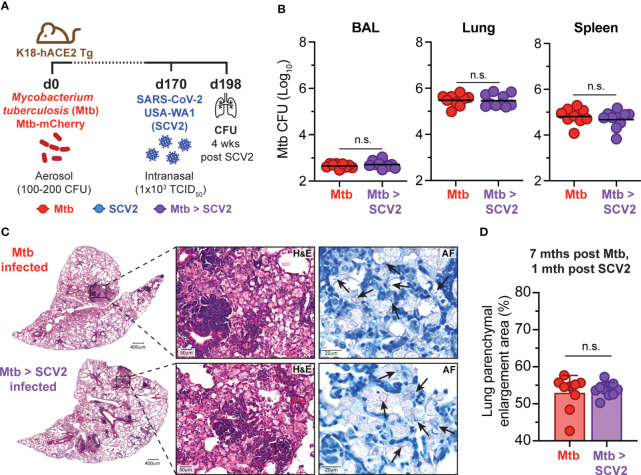
SCV2 co-infection does not exacerbate *Mtb* disease. **(A)** Schematic of experimental set-up where K18-hACE2 Tg mice were aerosol infected with 100 – 200 CFU *Mtb* (H37Rv-mCherry) 170 days before being intranasally infected with 1x10^3^ TCID_50_ SCV2 (USA-WA1/2020) or mock supernatant, mice were euthanized 1 month after SCV2 infection. **(B)** *Mtb* CFU in BALs, lungs and spleens (n= 9-10 per group, data combined from two independent experiments, geometric mean). **(C)** Representative H&E and AF staining of lung tissue from mice as described in **(A)** (arrows indicate examples of *Mtb* bacteria, scale bars indicate magnification) **(D)** Quantification of percentage of parenchymal enlargement from H&E shown in **C**) (n= 9-10 per group from two independent experiments, mean ± S.D., two-tailed Mann Whitney test). n.s. = not significant.

We and others have reported that concurrent or sequential infection with IAV and *Mtb* resulted in loss of bacterial control (17, 18). To ask whether SCV2 co-infection could equally compromise *Mtb* replication, K18-hACE2 Tg mice were first infected with *Mtb*. At a later stage of infection (day 170 post-*Mtb*) *Mtb*-infected mice and age-matched controls were then infected with a sub-lethal dose of USA-WA1/2020 SCV2 and monitored for 28 days after which bacterial burdens and lung pathology were assessed (**Figure 2A**). Importantly, SCV2 co-infection did not impact the bacterial burden of *Mtb* in bronchoalveolar lavage (BAL), lungs, or spleens (**Figure 2B**). Additionally, no differences were seen in lung pathology or bacterial localization as determined by H&E and AF staining (**Figure 2C**) or scoring of affected lung areas (**Figure 2D**).

**Figure 3:**
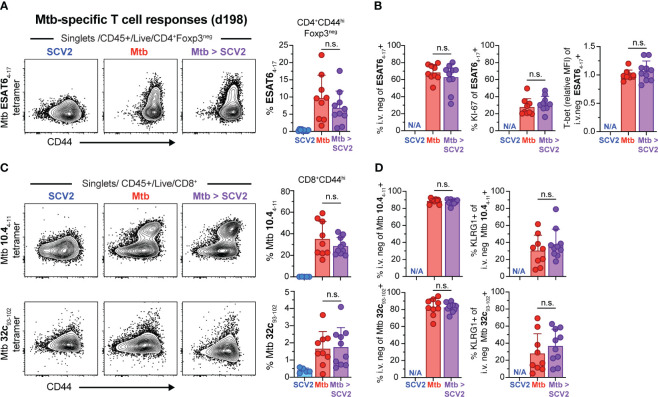
SCV2 co-infection does not negatively affect *Mtb*-specific CD4^+^ or CD8^+^ T cells. Example FACS plots and summary data from the lungs of mice described in **Figure 2A**. **(A)** Example FACS plots of ESAT6_4-17_ MHC-II tetramer staining of CD4^+^ Foxp3^-^ cells and proportion of ESAT6_4-17_-specific cells within activated CD44^hi^CD4^+^Foxp3^-^ T cells **(B)** Quantification of ESAT6_4-17_ tetramer-positive cells that are recruited into the lung parenchyma (CD45 i.v.^neg^), positive for Ki-67 and relative expression intensity (geometric mean fluorescent intensity, MFI) of T-bet in lung resident ESAT6_4-17_ tetramer-positive CD4^+^ T cells. **(C)** Example FACS plots of *Mtb* TB10.4_4-11_ (top) and *Mtb* 32c_93-102_ (bottom) MHC-I tetramer staining of CD8^+^ T cells and proportion of *Mtb* TB10.4_4-11_ or *Mtb* 32c_93-102_ -specific CD8^+^ T cells gated on activated CD44^hi^CD8^+^ T cells **(D)** Quantification of *Mtb* TB10.4_4-11_ (top) and *Mtb* 32c_93-102_ (bottom) tetramer-positive cells recruited into the lung parenchyma (CD45 i.v.^neg^) and their expression of KLRG1 (N/A= not applicable, n= 9-10 per group, data combined from 2 independent experiments, mean ± S.D., two-tailed Mann Whitney test). n.s. = not significant.

Next, we asked whether co-infection with SCV2 at 16 weeks post *Mtb* infection could negatively impact the existing *Mtb-*specific CD4^+^ or CD8^+^ T cell responses. When we quantified *Mtb* (ESAT6_4-17_)-specific CD4^+^ T cells *via* MHC-II tetramer straining one month following co-infection with SCV2, the frequency of antigen-specific CD4^+^ T cells was unchanged between mice infected solely with *Mtb* or those co-infected with SCV2 (**Figure 3A**). There were also no differences in the proportion of lung parenchyma-residing ESAT6_4-17_-specific CD4^+^ T cells, as assessed by lack of intravenous CD45 staining (i.v.^neg^) (32), nor in the expression of Ki-67 or levels of the transcription factor T-bet on those cells (**Figure 3B**). Likewise, when we examined *Mtb*-specific CD8^+^ T cell responses the overall abundance (**Figure 3C**) and proportion of parenchymal or KLRG1-expressing cells within *Mtb* TB10.4_4-11_ and *Mtb* 32c_93-102_ MHC-I tetramer positive CD8^+^ T cells were similar between co-infected lungs and lung from mice infected with only *Mtb* (**Figure 3D**). Together these data suggest that the pre-existing pulmonary *Mtb*-specific CD4^+^ and CD8^+^ T cell responses are not negatively impacted over the course of 4 weeks following SCV2 co-infection.

**Figure 4:**
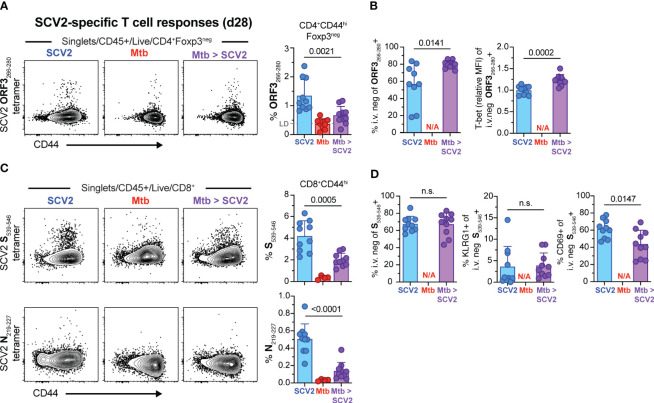
SCV2 co-infection of *Mtb* infected mice results in decreased SCV2-specific CD4^+^ and CD8^+^ T cells in the lungs 4 weeks later. Example FACS plots and summary data from the lungs of mice described in **Figure 2A**. **(A)** Example FACS plots of SCV2 ORF3_266-280_ MHC-II tetramer staining of CD4^+^ Foxp3^-^ cells and proportion of ORF3_266-28_-specific cells within activated CD44^hi^CD4^+^Foxp3^-^ T cells 4 weeks after SCV2 infection of naïve (blue) or 7-month *Mtb*-infected mice (purple). LD = Limit of Detection is indicated based on non-specific tetramer binding in *Mtb* only infected groups (red) **(B)** Quantification of ORF3_266-280_ tetramer-positive cells residing in lung parenchyma (CD45 i.v.^neg^) and relative expression intensity (geometric mean fluorescent intensity, MFI) of T-bet in lung resident ORF3_266-280_ tetramer-positive CD4^+^ T cells. **(C)** Example FACS plots of SCV2 S_539-546_ (top) and SCV2 N_219-227_ (bottom) MHC-I tetramer staining of CD8^+^ T cells and proportion of S_539-546_- or N_219-227_- specific CD8^+^ T cells gated on activated CD44^hi^CD8^+^ T cells **(D)** Quantification of SCV2 S_539-546_ tetramer-positive cells recruited into the lung parenchyma (CD45 i.v.^neg^) and their expression of KLRG1 and CD69. (N/A= not applicable, n= 9-10 per group, data combined from 2 independent experiments, mean ± S.D., two-tailed Mann Whitney test). n.s. = not significant.

We next measured SCV2 antigen-specific T cell responses 4 weeks after SCV2 infection in mice with and without an underlying *Mtb* infection using SCV2-specific tetramers (33). To measure SCV2 specific CD4^+^ T cells we utilized an ORF3A_266-280_ MHC-II I-A^b^ tetramer and 4 weeks after sub-lethal infection detected approximately 1-2% of effector CD4 T cells that stained positive for the reagent (blue symbols) by flow cytometry, compared to less than 1% in *Mtb* co-infected mice (purple symbols) and 0.5% non-specific staining background in SCV2 unexposed animals (red symbols) (**Figure 4A**). Thus the overall frequency of ORF3_266-280_ specific CD4^+^ T cells was significantly reduced in the lungs of co-infected mice compared to their SCV2-only counterparts. Conversely, proportionately more ORF3_266-280_ specific cells were residing in the lung parenchyma (i.v.^neg^) and expressed a small but significant increase in T-bet expression (**Figure 4B**). Importantly, both the SCV2 S_539-546_-specific and SCV2 N_219-227_-specific CD8^+^ T cell responses were significantly reduced in mice with an underlying *Mtb* infection compared to SCV2 alone (**Figure 4C**). Furthermore, while the overall proportion of i.v.^neg^, KLRG1-expressing S_539-546_-specific CD8^+^ T cells was the same regardless of *Mtb* infection status, the frequency of tissue-resident memory (T_RM_, CD69^+^) T cells, was significantly reduced within that fraction in co-infected lungs (**Figure 4D**). Thus, ongoing *Mtb* infection resulted in a significant reduction in the magnitude of the pulmonary SCV2 S_539-546_ specific T_RM_ response.

### Underlying *Mtb* infection reduces initial SCV2 viral burden independent of IFN-I

**Figure 5:**
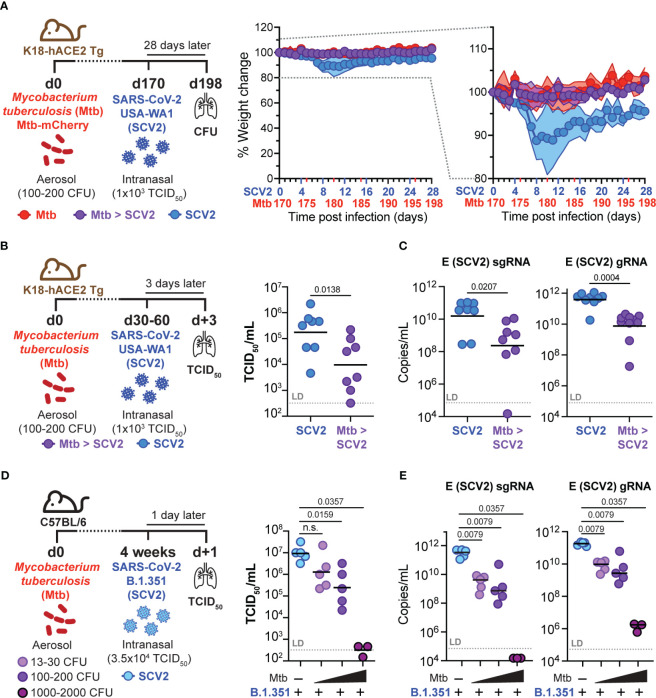
Pre-existing *Mtb* infection lowers early SCV2 viral burden in an *Mtb* dose-dependent manner. **(A)** Left: Schematic of experimental set-up where K18-hACE2 Tg mice were infected with *Mtb* 170 days before being intranasally infected with 1x10^3^ TCID_50_ SCV2 (USA-WA1/2020) or mock supernatant, mice were euthanized 28 days after SCV2 infection. Middle: Weight loss of SCV2 infected K18-hACE2 Tg mice with (purple) or without (blue) underlying *Mtb* infection Right: Selected range of weight change curve to highlight differences in weight loss between SCV2 only and co-infected groups (n= 9-10 per group pooled from 2 independent experiments, mean ± SD as traveling error bars). **(B)** Left: Schematic of experimental set-up where K18-hACE2 Tg mice were infected with *Mtb* by aerosol exposure 1-2 months before infection with 1x10^3^ TCID_50_ SCV2 (USA-WA1/2020), mice were euthanized 3 days after SCV2 infection. Right: SCV2 viral load in lungs as measured by TCID_50_ and **(C)** qPCR for the sub-genomic (sg) or genomic (g) SCV2 E gene (n= 8 per group, data combined from two independent experiments, geometric mean, two-tailed Mann Whitney test, LD= limit of detection). **(D)** Left: Schematic of experimental set-up where C57Bl/6 WT mice were infected with various doses of *Mtb* (H37Rv-mCherry) by aerosol exposure 4 weeks before being intranasally infected with 3.5x10^4^ TCID_50_ SCV2 (B.1.351), mice were euthanized 1 day later. Right: Viral loads in lung as measured by TCID_50_ on Vero E6 cells and **(E)** qPCR for the SCV2 E gene sgRNA and gRNA (right) (n= 3 – 5 per group from one experiment representative of two independent experiments, geometric mean, statistical significance calculated by two-tailed Mann Whitney test, LD= limit of detection). n.s. = not significant.

Considering that antigen burden can directly impact T cell expansion and memory development (34), we asked whether the decrease in SCV2-specific CD8^+^ T_RM_ frequency 4 weeks after SCV2 infection in *Mtb*-infected mice was caused by a change in the initial SCV2 viral burden. We suspected that viral loads were reduced in co-infected mice as susceptible K18-hACE2 Tg mice lost 10% of their pre-SCV2 infection body weight 5 – 8 days after SCV2 infection but did not lose any weight if they were also infected with *Mtb* (**Figure 5A**). To determine whether SCV2 viral titers were reduced in the lungs of co-infected mice, we infected K18-hACE2 Tg mice with USA-WA1/2020 either with or without underlying *Mtb* infection and collected lungs at 3 days post SCV2 infection, which is early enough to determine viral loads. Indeed, ongoing *Mtb* infection reduced SCV2 lung viral titers by 1-2 logs at 3 days post-infection as measured by both TCID_50_ assay on Vero E6 cells (**Figure 5B**) and quantitative PCR (qPCR) to measure the number of copies of the SCV2 E gene in both its actively replicating (sub-genomic, sgRNA) and typical (genomic, gRNA) conformations (35) (**Figure 5C**). Recognizing that the K18-hACE2 model has changes in viral tropism due to the artificial nature of the hACE2 transgene expression (36), we utilized a SCV2 variant of concern (VOC, beta variant, B.1.351), which carries an asparagine to tyrosine substitution at amino acid 501 of the spike protein, allowing binding to murine ACE2 and establishment of transient SCV2 infection in wild type (WT) C57Bl/6 mice (37, 38). Strikingly, we observed a significant reduction in B.1.351 SCV2 viral titers in the lungs of *Mtb*-infected C57Bl/6 mice as early as 1 day post SCV2 infection, and the magnitude of viral restriction correlated with increasing *Mtb*-infectious dose, with no replicating virus detectable in the lungs of mice that previously received a high dose of *Mtb* (1000 - 2000 CFU) (**Figures 5D, E**). Taken together, these results suggest that an underlying pulmonary *Mtb* infection restricts early viral replication, leading to a reduction in overall viral antigens and a decrease in the magnitude of the T cell and T_RM_ response.

**Figure 6:**
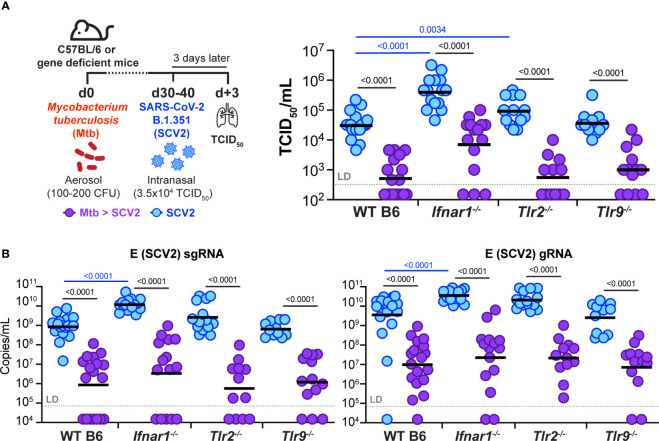
Underlying *Mtb* infection reduces SCV2 viral burden independent of IFNAR1, TLR2, or TLR9. **(A)** Left: Schematic of experimental set-up where C57Bl/6 WT, *Ifnar1* ^-/-^, *Tlr2* ^-/-^ or *Tlr9* ^-/-^ mice were infected with *Mtb* 30-40 days prior to being intranasally infected with 3.5x10^4^ TCID_50_ SCV2 (B.1.351), mice were euthanized 3 days after SCV2 infection. Right: SCV2 viral load in lungs as measured by TCID_50_ on Vero E6 cells. **(B)** SCV2 viral loads in lungs as measured by qPCR for the SCV2 E sgRNA (left) or gRNA (right) (n= 11-19 per group, data combined from four independent experiments, geometric mean, two-tailed Mann Whitney test (only significant p values shown), LD= limit of detection; significant differences are indicated by blue comparisons between SCV2 only groups (blue), significant differences are indicated by black comparisons between SCV2 (blue) and coinfected groups (purple).

Because our findings showed restriction of viral replication as early as one day after SCV2 infection in a *Mtb* dose-dependent manner, we speculated that *Mtb*-driven innate inflammation, alongside induction of antiviral interferons, may mediate the observed protective effects. *Mtb* carries several pathogen-associated molecular patterns (PAMPs) that activate pattern recognition receptors (PRRs), including TLR2 and TLR9 (39). TLR activation leads to production of several inflammatory cytokines, including IFN-I. Due to the potent antiviral nature of IFN-I (8), we next examined whether *Mtb* sensing *via* TLR2 or TLR9 or a *Mtb*-driven IFN-I response were required for SCV2 restriction in *Mtb*-infected mouse lungs. We infected mice deficient in the IFNα receptor 1 (IFNAR1, *Ifnar1* ^-/-^), TLR2 (*Tlr2^-/-^*), or TLR9 (*Tlr9^-/-^*) with *Mtb* and 1-2 months later with B.1.351 SCV2 (**Figure 6A**). Of note, without underlying *Mtb* infection (blue symbols), *Ifnar1* ^-/-^ mice displayed a 1.0 – 1.5 log significant increase in viral titers at three days after B.1.351 SCV2 infection compared to WT mice as measured by both TCID_50_ (**Figure 6A**) and qPCR (**Figure 6B**). SCV2-infected *Tlr2^-/-^* mice had a significant increase in viral titers when measured by TCID_50_ (**Figure 6A**) but not when assessed by qPCR (**Figure 6B**). *Tlr9^-/-^* mice showed no differences in lung viral titers (**Figures 6A, B**). Importantly and irrespective of these baseline increases in viral titers, we consistently observed a 1.5 – 2.0 log reduction in SCV2 viral loads in lungs of mice with an underlying *Mtb* infection, regardless of their expression of TLR2, TLR9, or IFNAR1 (**Figures 6A, B**). These results indicate that *Mtb*-induced restriction of SCV2 is not dependent solely on TLR2, TLR9 or IFN-I signaling and likely is a consequence of multiple innate inflammatory immune alterations during *Mtb* infection compared to the lungs of immunologically naïve mice.

## Discussion

To investigate the immunological consequences of *Mtb* and SCV2 interactions we have utilized various murine co-infection models. We have shown that *Mtb*-infected mice that have recovered from a prior SCV2 infection showed no significant changes in *Mtb* bacterial burden or lung pathology. In addition, SCV2 co-infection of chronically *Mtb*-infected mice did not negatively impact bacterial control, lung pathology, or existing *Mtb*-specific T-cell responses. Importantly, using these models we have also demonstrated that early SCV2 replication is dampened in the lungs of *Mtb*-infected K18-hACE2 Tg and C57Bl/6 mice compared to mice without an underlying *Mtb* infection. This protective effect was *Mtb* dose-dependent, prevented SCV2-induced weight loss, and was associated with lower SCV2-specific memory T cell responses compared to mice infected only with SCV2. Our observations agree with previous data published by Rosas-Mejia and colleagues who first showed that concurrent co-infection with *Mtb* and SCV2 in both K18-hACE2 Tg and WT C57Bl/6 mice reduced SCV2 viral titers but did not affect *Mtb* bacterial loads (31). The Rosas-Meija study also showed that co-infection of mice with *Mtb* and SCV2 altered cytokine production and the abundance of immune cell subsets as determined by single cell RNA sequencing (scRNASeq) at 4 – 7 days post SCV2 infection compared to mice infected with either pathogen individually (31). Compared to lungs from SCV2 only mice, co-infected lungs had elevated IFNγ protein, increased Tumor Necrosis Factor (*Tnf*) transcript, reduced IFN-induced protein with tetratricopeptide repeats 2 (*Ifit2)* and *Ifit3* transcripts, and scRNASeq indicated an increased proportion of B cells and a reduced frequency of CD8^+^ T cells. Our data adds that these perturbations do not affect the *Mtb*-specific T cell response, and instead may contribute to the reduction in SCV2-specific T cell responses that we have observed at one month post SCV2 co-infection. In addition, Hildebrand et al. reported a significant decrease in SCV2 viral titers at four days post SCV2 infection of *Mtb*-infected mice (30), but interestingly, they saw a significant increase in splenic *Mtb* loads while pulmonary *Mtb* burdens were not significantly changed (16). The discrepancy in bacterial replication seen by Hildebrand et al. may be due to their use of the Erdmann strain of *Mtb* compared to the H37Rv laboratory strain used both herein and in the Rosas Mejia study, suggesting the outcome of *Mtb* and SCV2 co-infection may be modulated by differences in the strain of *Mtb*. In turn, it is also likely that the strain and variant of SCV2 itself can influence disease during an underlying *Mtb* infection. Future studies directly comparing diverse strains of *Mtb* and SCV2 in mouse coinfection models would advance our understanding of how virulence factors expressed by each pathogen contribute to the overall outcome of both diseases.

The inability of SCV2 to increase mycobacterial load and lung pathology in mice subsequently infected with *Mtb* contrasts with our previous experiments with sequential IAV and *Mtb* infections (17). SCV2 infection likely engages immune pathways differently in both quantity and/or quality compared to IAV, such as IFN-I production. Others have shown through *in vitro* infection of a human airway epithelial cell line susceptible to both viruses that IAV was a more potent inducer of IFN activity (as measured by STAT1 phosphorylation) than USA-WA1/2020 SCV2 (40). Additionally, COVID-19 patients hospitalized with pneumonia have comparatively low and delayed production of IFN-I when contrasted with severe IAV pneumonia patients (41). As IFN-I has a detrimental impact on *Mtb*-driven disease outcomes in mice and humans (17, 42–48), this raises the possibility that SCV2 may not be able to exacerbate *Mtb* infection due to the induction of a weaker IFN response compared to IAV infection. Future studies must systematically address commonalities and differences between the long-term impacts of IAV or SCV2 infection on the lung microenvironment and subsequent respiratory immune responses to secondary infections. Our data also do not exclude the possibility that *Mtb* burden may be altered at timepoints different from those tested here or whether SCV2 co-infection, similar to IAV co-infection (17, 18), can alter the mortality of *Mtb*-infected animals.

Using flow cytometric analyses, we investigated the impact of SCV2 infection 16 weeks post *Mtb* infection on existing *Mtb*-specific T cell responses, however no reduction was detected compared to mice infected with *Mtb* alone. Other studies where viruses were administered within the first two weeks after *Mtb*-infection (17, 18, 49), a critical time during which initial T cell priming to *Mtb* antigens occurs (50), have reported dampened *Mtb*-specific T cell responses and increased susceptibility to *Mtb*. We intentionally did not explore SCV2 co-infection within the first two weeks after *Mtb* infection because we wanted to mimic the most common clinical scenarios (i.e., individuals who had recovered from a previous SCV2 infection or individuals with a latent, underlying *Mtb* infection). While isolated *Mtb* components such as those present in Complete Freund’s Adjuvant can serve as adjuvants to amplify adaptive responses to specific peptide antigens (51, 52), any potential adjuvant effect caused by infection with live *Mtb* bacteria here, was unable to boost antigen-specific T cell responses to SCV2. In contrast, we showed that ongoing *Mtb* infection reduced the frequency of SCV2-specific CD8^+^ T_RM_, which was likely due to strongly enhanced early innate viral control in *Mtb* infected mice. We cannot, however, completely rule out that the underlying *Mtb* infection additionally influenced the priming, expansion, contraction, and migration of SCV2-specific T cells into the lungs of co-infected mice; Therefore, further studies are needed to address how simultaneous infection with SCV2 and *Mtb* impacts the priming of adaptive immune responses and disease outcomes for each pathogen.

The *Mtb* infection-mediated suppression of SCV2 replication in our studies was apparent as early as one day post SCV2 exposure. As such, we propose that underlying *Mtb* infection enhances early anti-viral innate immunity in the lung. In contrast to the detrimental role IFN-I plays in *Mtb* infection, IFN-I is a critical family of cytokines promoting innate anti-viral immunity (8). However, our data suggest that IFN-I signaling did not mediate the increased viral control in *Mtb*-infected lungs, as we showed that *Ifnar1* ^-/-^ mice had a similarly reduced SCV2 burden as WT mice when previously infected with *Mtb*. Nevertheless, we also showed that IFNAR1-deficient mice had a higher viral load at 3 days post-infection independently of *Mtb* infection status, in line with previously published *in vivo* data that support IFN-I-dependent control of SCV2 replication in mice (53–57) and hamsters (58, 59). Genes induced by IFN-I signaling (Interferon Stimulate Genes, ISGs) overlap significantly, but not completely with genes induced by IFNγ (IFN-II) and IFNλ (IFN-III) (60, 61), suggesting that production of any family of IFNs may have similar effects during SCV2 infection. Several studies utilizing mice deficient in IFNγ signaling have demonstrated a direct role for IFN-II in restriction of SCV2 (62–64). Moreover, IFNγ plays a central role in SCV2 restriction following intravenous infection of mice with *Mycobacterium bovis* bacille Calmette-Guérin (BCG), an attenuated relative of *Mtb* (62, 65). Studies of IFNλ in SCV2-infected mice indicate that the IFN-III response can also restrict viral replication in the mouse lung (66). However, IFNλ signaling-deficient hamsters did not exhibit a similar defect in viral control (67). Thus, while *Mtb*-driven control of early viral replication occurred independently of IFN-I in our studies, it is possible that IFN-III and/or IFN-II, the latter of which is highly induced after mycobacterial infections in mice and is responsible for reduced SCV2 burden following infection with BCG (65), may contribute to the anti-viral state in *Mtb*-infected lungs.

Finally, because early viral restriction was *Mtb*-infection dose-dependent, we explored whether suppression of viral replication in *Mtb*-infected mice was mediated through mycobacterial sensing by TLR2 or TLR9. In addition to recognition of mycobacterial ligands, a role for TLR2 in recognizing the envelope (E) protein of SCV2 leading to innate viral control has been previously reported (68), and we show here that TLR2-deficient mice indeed displayed higher TCID_50_ viral titers in their lungs three days after infection with B.1.351 SCV2 in the absence of *Mtb* infection. Nevertheless, neither TLR2 nor TLR9 signals were individually responsible for suppressing SCV2 replication in the lungs of *Mtb* co-infected C57Bl/6 mice. Our data do not exclude the possibility that multiple TLRs may act in concert to induce a SCV2-suppressive immune environment during *Mtb* infection, and further functional studies are needed to uncover the complex immunological mechanisms responsible for increased innate anti-viral immunity. These mechanisms may include but are not limited to down-regulation of viral entry receptors, enhanced antiviral activation of lung epithelial cells, trained innate immunity or modulation of the innate immune cell milieu in the lung (64, 65, 69–71).

Together, our data suggest that, in the K18-hACE2 Tg mouse model, infection with the ancestral strain of SCV2 does not exacerbate ongoing or subsequent *Mtb* infection at the timepoints tested. Further studies utilizing different strains of mice, *Mtb* strains and SCV2 variants that explore the entire course of infection would further strengthen these observations. While our data do not rule out the possibility of immunological influences in the exacerbation of TB or COVID-19 in co-infected humans they do point to the impact of sociological and healthcare disruptions as more significant factors underlying the reported increase in TB mortality rates during the COVID-19 pandemic.

## Methods

### Mice

K18-hACE2 Tg hemizygous transgenic mice (B6.Cg-Tg(K18-ACE2)2Prlmn/J; JAX stock #034860) (36), were purchased from Jackson Laboratories (Bar Harbor, ME). C57Bl/6 mice or C57Bl/6 mice expressing a *Foxp3*-GFP reporter (C57BL/6-Foxp3^tm1Kuch^) (72) were used as wild type C57Bl/6 controls. *Foxp3*-GFP mice and *Ifnar1* KO mice (B6-[KO]IFNa/bR1) (73) were obtained through a supply contract between NIAID and Taconic Farms. *Tlr2* KO mice (74) and *Tlr9* KO mice (75) were originally generated by the laboratory of Dr. Shizuo Akira (Osaka University, Japan) and were kind gifts of Dr. Alan Sher (NIH/NIAID) and Dr. Giorgio Trinchieri (NIH/NCI) respectively. All mouse strains were confirmed to be on a C57Bl/6 background by genetic background analysis submitted through Transnetyx and performed by Neogen using the MiniMUGA platform. Both male and female mice were used and were 8-16 weeks old at the onset of experiments and mice within experiments were age and sex matched. All animals were bred and maintained in an AAALAC-accredited ABSL2 or ABSL3 facility at the NIH and experiments were performed in compliance with an animal study proposal approved by the NIAID Animal Care and Use Committee.

### *Mtb* infection of mice

Aerosol infections of mice with H37Rv-mCherry (50-200 CFU, or as indicated in figure legends) were carried out in a Glas-Col whole-body inhalation exposure system as previously described in detail (76). Briefly, to quantify *Mtb* CFU, lung or spleen homogenates, BALF or inocula were serial-diluted in PBS + 0.1% Tween-80 and plated on Middlebrook 7H11 agar (Sigma Aldrich) supplemented with oleic acid-albumin-dextrose-catalase (OADC) for 3 weeks at 37°C before colonies were counted.

### SARS-CoV-2 infection of mice

SARS-CoV-2 hCoV-19/USA-WA1/2020 (Pango lineage A, GISAID reference: EPI_ISL_404895.2) (USA-WA1/2020) and SARS-CoV-2/human/ZAF/KRISP-K005325/2020 beta variant of concern (Pango lineage B.1.351, GISAID reference: EPI_ISL_678615) (B.1.351) were obtained from BEI resources (NIAID, NIH). Viral stocks were generated by infection of Vero cells (CCL-81, American Type Culture Collection) without (USA-WA1/2020) or with (B.1.351) stable expression of TMPRSS2 (77) at a multiplicity of infection of 0.01 for 48hrs. Cell culture media was harvested and centrifuged at 3500 x g, pooled, aliquoted, and stored at -80°C until use. Virus stocks were sequenced using the Illumina platform; USA-WA1/2020 was consistent with the reference sequence MN985325.1 except for H655Y in S, S6L in E, T7I in M, and S194T in N; B.1.351 was consistent with reference sequence MZ376663.1. Mice were anesthetized with isoflurane and infected intranasally with 35µL inoculum containing 1.0x10^1^ - 1.0x10^3^ TCID_50_ USA-WA1/2020 or 3.5x10^4^ TCID_50_ B.1.351. Inoculum was quantified by TCID_50_ assay in Vero E6 cells (CRL-1586; American Type Culture Collection).

### Viral quantification by TCID_50_ assay

2.5x10^4^ Vero E6 cells were seeded in 100μL DMEM + 10% FCS per well of 96-well tissue culture cluster plates, incubated at 37°C + 5% CO_2_ for 16-24 hrs and washed twice with 100μL DMEM + 2% FCS before the assay was conducted. After harvesting lungs from mice, the inferior lobe, post-caval lobe and left lung were homogenized in 600μL PBS using 2.7mm glass beads on a Precellys tissue homogenizer (Bertin Instruments) before dilution with PBS to a final volume of 1.7mL. Viral titers were determined by performing 10-fold serial dilutions of homogenates in DMEM + 2% FCS in quadruplicate, then plating 100μL serial-diluted homogenate and 100μL DMEM + 2% FCS on washed Vero E6 cells and incubating at 37°C + 5% CO_2_ for 96 hours. TCID_50_ was measured by removing supernatants and staining wells with crystal violet before scoring for cytopathic effect and calculation using the Reed–Muench method.

### RNA extraction and quantitative PCR of viral genomes

For RNA extraction, the superior lobe from each mouse was placed in RNAlater (Invitrogen) and stored at −80°C. RNAlater-stabilized lung lobes were thawed at RT for 20 min, then homogenized in RLT Plus buffer with β-mercaptoethanol (QIAGEN). Total RNA was then isolated from the RLT-homogenized tissue using the RNeasy Plus Mini Kit (QIAGEN), including on-column DNase treatment using the RNase-Free DNase set (QIAGEN) following the manufacturer’s instructions and eluted in 60µL RNAse-free water. SCV2 genome copy quantitation was performed in duplicate from 2.5uL of eluted RNA per reaction using the Taqpath 1-step RT-qPCR Master Mix (Thermo) as described by the manufacturer. The SCV2 E gene in both typical (genomic, gRNA) and actively replicating (sub-genomic, sgRNA) conformations (35) was detected using primers at 500nM as follows: E (genomic) Forward (5’- ACAGGTACGTTAATAGTTAATAGCGT-3’), E (sub-genomic) Forward (5’- CGATCTCTTGTAGATCTGTTCTC-3’), E (genomic and sub-genomic) Reverse (5’- ATATTGCAGCAGTACGCACACA -3’) and the probe for both E genomic and sub-genomic reactions was used at 125nM (5′- (FAM)-ACACTAGCCATCCTTACTGCGCTTCG-(3IABkFQ) -3′). Cycling conditions: Initial: 25°C for 2 min, 50°C for 15 min, and 95°C for 2 min, Cycling: 95°C for 3 sec, 60°C for 30 sec, for 40 cycles. Copy number was calculated based on standard curves generated for each RT-qPCR run, with SCV2 RNA standard of known quantity and eleven 5-fold dilutions run in duplicate (78).

### Cell isolation for flow cytometry

Lungs from infected mice were dissociated using a GentleMACS dissociator (Miltenyi Biotec) in digestion buffer comprised of 0.33mg/mL Liberase TL (Roche), 7U/mL benzonase (Sino Biological), 10µM cytochalasin D (Sigma-Aldrich) and 200µg/mL hyaluronidase (Sigma-Aldrich) followed by 30 - 45 minutes at 37°C. Digested lung was fully dispersed by passage through a 100µm pore size cell strainer and an aliquot was removed for bacterial CFU measurements when needed. Isolated cells were stained with MHC-II tetramers for 40min at 37°C in complete RPMI with 1mM aminoguanidine (Sigma-Aldrich), 100nM dasatinib (Cayman Chemical), 3µg/mL brefeldin A (ThermoFisher) and 2µM monensin (ThermoFisher). Cells were then washed and stained with MHC-I tetramers, surface antibodies and Molecular Probes LIVE/DEAD Fixable Near-IR Dead Cell Stain Kit (ThermoFisher) for 20 min at 4°C before permeabilization and fixation using eBioscience™ Foxp3 Transcription Factor Staining Buffer Set (ThermoFisher) at 4°C overnight. Intracellular staining was performed in eBioscience™ Permeabilization Buffer (ThermoFisher) for 40 minutes at 4°C. Samples were acquired on a FACSymphony (BD Biosciences). FACS data were analyzed using FlowJo10 (Treestar). Antibodies were purchased from BioLegend, BD and ThermoFisher as follows: anti-CD45 (30-F11), anti-CD4 (GK1.5), anti-FoxP3 (FJK-16s), anti-CD8a (53-6.7), anti-CD44 (IM7), anti-Ki-67 (B56), anti-T-bet (4B10), anti-KLRG1 (2F1), anti-CD69 (H1.2F3). Tetramer reagents were obtained from the NIH Tetramer Core Facility as follows: *Mtb* ESAT6_4-17_ MHC-II I-A^b^ tetramer, *Mtb* TB10.4_4-11_ MHC-I H-2K^b^ tetramer, *Mtb* 32c_93-102_ MHC-I H2-D^b^ tetramer, SCV2 ORF3A_266-280_ MHC-II I-A^b^ tetramer, SCV2 S_532-546_ MHC-I H-2K^b^ tetramer, SCV2 N_219-227_ MHC-I H-2D^b^ tetramer.

### Histopathology

The middle right lung lobe from each mouse was fixed in 4% paraformaldehyde, transferred to 70% ethanol and paraffin-embedded before sectioning and mounting on glass slides for staining with hematoxylin and eosin (H&E) or the Kinyoun method for visualization of acid-fast (AF) mycobacteria. Stained slides were imaged by light microscopy on an Aperio Versa microscope (Leica Microsystems). Images were processed using QuPath v0.3.2 (Bankhead et al., 2017) and ImageJ v1.53t (NIH) for quantification and visualization as previously described (79).

### Statistical analyses

Statistical analyses were performed using GraphPad Prism v9.0 for Mac OS X (GraphPad Software). Each figure legend lists all the statistical details of experiments, including the statistical tests used. Data are expressed as mean ± SD. Significant differences are indicated by the p value in each figure.

## Data availability statement

The raw data supporting the conclusions of this article will be made available by the authors, without undue reservation.

## Ethics statement

The animal study was approved by National Institute of Allergy and Infectious Diseases IACUC. The study was conducted in accordance with the local legislation and institutional requirements.

## Author contributions

Conceptualization: PB and KM-B. Investigation: PB, EA, EC, AB, FT-J, CJ, KH and KM-B. Resources: CN, DB, RJ and KM-B. Data Analysis and Curation: PB, EA and KM-B. Data Visualization: PB, EA and KM-B. Writing – Original Draft: PB and KM-B, Writing – Review & Editing: all co-authors. Supervision: DB and KM-B. Funding Acquisition: RJ, DB, and KM-B. All authors contributed to the article and approved the submitted version.
